# Network Pharmacology Prediction and Molecular Docking-Based Strategy to Explore the Potential Mechanism of Gualou Xiebai Banxia Decoction against Myocardial Infarction

**DOI:** 10.3390/genes15040392

**Published:** 2024-03-22

**Authors:** Wei-Lu Wang, Yan Chen

**Affiliations:** Faculty of Medicine, Macau University of Science and Technology, Praia Park Block R Coloane Macau, Macau 999078, China; 3220006902@student.must.edu.mo

**Keywords:** network pharmacology, molecular docking, Gualou Xiebai Banxia decoction, targets

## Abstract

The aim of this study was to investigate targets through which Gualou Xiebai Banxia decoction aids in treating myocardial infarction (MI) using network pharmacology in combination with molecular docking. The principal active ingredients of Gualou Xiebai Banxia decoction were identified from the TCMSP database using the criteria of drug-likeness ≥30% and oral bioavailability ≥0.18. Interactions and pathway enrichment were investigated using protein–protein interaction (PPI) networks and Gene Ontology (GO) and Kyoto Encyclopedia of Genes and Genomes (KEGG) enrichment analysis, respectively. Active component structures were docked with those of potential protein targets using AutoDock molecular docking relative softwares. HIF1A was of particular interest as it was identified by the PPI network, GO and KEGG pathway enrichment analyses. In conclusion, the use of network pharmacology prediction and molecular docking assessments provides further information on the active components and mechanisms of action Gualou Xiebai Banxia decoction.

## 1. Introduction

Myocardial infarction (MI) results from arterial plaque accumulation leading to reductions in blood flow, reduced oxygen and damage to the heart muscles [1,2,3]. MI symptoms include chest pain, heartbeat abnormalities, shortness of breath and nausea, amongst others [2,4]. If these symptoms are not treated rapidly, MI can lead to death [5,6]. It is thus important to prevent and alleviate the symptoms of MI to save patients’ lives.

Traditional Chinese medicine (TCM) has been used for many thousands of years in China [7]. The Gualou Xiebai Banxia decoction is an herbal formula that was first described in the “Synopsis of the Golden Chamber” by Zhang Zhongjing in the Eastern Han Dynasty [8]. It is composed mostly of Gualou, Xiebai and Banxia [9]. For centuries, Gualou and Xiebai have been used for the clinical treatment of cardiovascular diseases (CVDs), including MI, heart failure and arrhythmia [10,11]. Extracts of Gualou (*Trichosanthes kirilowii Maxim*) and Xiebai (*Allium macrostemon Bge*) have been found to improve cardiac function and hemodynamics and have been shown to be effective in treating ischemia/reperfusion injuries and MI [12,13,14]. Banxia (*Pinellia pinellia*), likewise, has significant therapeutic actions in CVD [14]. The Gualou Xiebai Banxia decoction is a commonly prescribed TCM for the treatment of CVD, including MI [15]. Furthermore, Gualou Xiebai Banxia decoction has been found to protect against type II diabetes (T2DM) treatment with acute myocardial ischemia (AMI) (T2DM-AMI) by attenuating oxidative stress (OS) and apoptosis via phosphatidyl inositol 3-kinase (PI3K)/serine/threonine protein kinase B (Akt)/endothelial nitric oxide synthase (e-NOS) signaling [15]. Specifically, Gualou Xiebai Banxia decoction is reported to upregulate PI3K, Akt and e-NOS mRNA and protein in T2DM-AMI rats, leading to protection against T2DM-AMI [15]. Although the Gualou Xiebai Banxia decoction has been demonstrated to have a specific protective effect against MI, its mechanism of action and the pathways involved still require further study.

Network pharmacology was introduced by Hopkins in 2007 [16,17] and predicts drug–target interactions based on the structures of the interacting molecules and the effectiveness of the drug’s action. This approach takes into account the multiple interactions occurring within the body and constructs a network between the drug, the molecular target and the disease, together with an analysis of the genes associated with the disease [18]. An understanding of the signaling and regulatory pathways involved in drug action can both improve the efficacy of the drug and reduce its side effects, thus ultimately improving the success of clinical trials and saving costs [19]. Network pharmacology uses multiple levels of analysis, including omics datasets and in silico simulations, to analyze the mechanisms underlying drug effects. It is, thus, an ideal approach for analyzing the complexities of TCM [20]. It is worth mentioning the relevance of molecular docking to drug research as it simulates the recognition process between molecules and predicts both the binding mode and the strength of the interaction between molecules and receptors through a scoring function. In recent years, molecular docking has contributed significantly to the field of computer-aided medical research.

**Figure 1:**
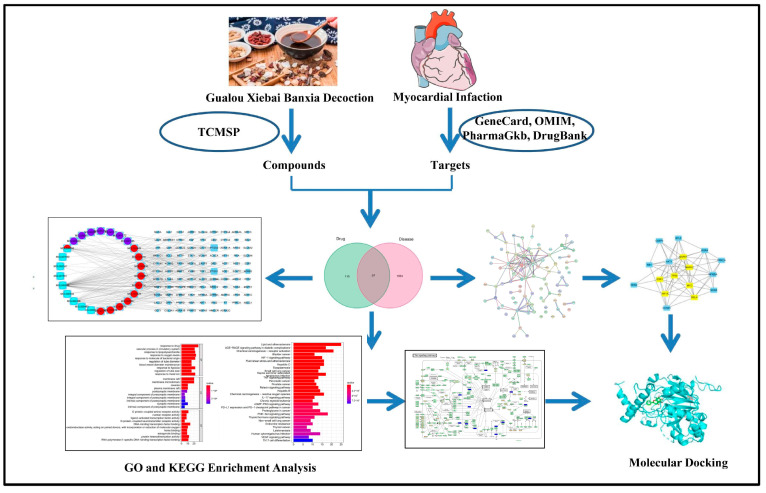
A flow chart depicting the whole framework based on network pharmacology and molecular docking.

Here, we used network pharmacology to explore the main components and molecular biological mechanisms of Gualou Xiebai Banxia decoction for the treatment of MI. Molecular docking was then used to verify the predicted interactions between the identified active components of Gualou Xiebai Banxia decoction and their molecular targets. The combination of molecular docking and network pharmacology provides a means of effectiveness for studying the action of Gualou Xiebai Banxia decoction, as well as a reference for follow-up research. Figure 1 shows a flow chart of the study.

## 2. Materials and Methods

### 2.1. Identification of the Active Components of Gualou Xiebai Banxia Decoction and Their Target Genes

The Traditional Chinese Medicine Systems Pharmacology Database and Analysis Platform (TCMSP) (TCMSP) (https://tcmsp-e.com/tcmsp.php, accessed on 1 January 2023) is a unique tool that can be used to investigate the active components and target genes of TCM [21]. Because the Gualou Xiebai Banxia decoction is composed mostly of “gualou”, “xiebai” and “banxia”, the TCMSP database was searched using the search terms “gualou”, “xiebai” or “banxia” followed by the screening criteria of oral bioavailability (OB) ≥30% and drug-likeness (DL) ≥0.18 [22,23]. The filtered results led to the compilation of the “gualou_ingredients.txt”, “xiebai_ingredients.txt” and “banxia_ingredients.txt” files. Similarly, using the “Related targets” column, we obtained three tables of TCM components and targets, leading to the complilation of the “gualou_targets.txt”, “xiebai_targets.txt” and “banxia_targets.txt” files. The files were then sorted and merged using Perl language to form an “allTargets.txt” file that included information on both ingredients and targets.

To incorporate the TCM ingredients and gene symbols in one table, the potential protein targets and gene abbreviations (gene symbols) were investigated using the UniProt database (https://www.uniprot.org/, accessed on 1 January 2023). Here, we chose “human” as the “popular organism” and “reviewed” under “filter by”. The filtered table was downloaded and copied into a single ann.txt file. This file was then integrated with the “allTargets.txt” file using Perl, resulting in the “allTargets symbol.txt” file.

### 2.2. Identification of Potential MI-Related Targets

The search term “myocardial infarction” was used in the OMIM (https://www.OMIM.org/, accessed on 1 January 2023), GeneCards (https://www.genecards.org/, accessed on 1 January 2023), DrugBank (https://www.DrugBank.ca, accessed on 1 January 2023) and PharmGkb (https://www.PharmGkb.org/, accessed on 1 January 2023) databases. In the GeneCards database, we selected potential targets with “Relevance Scores” greater than or equal to 1 for further investigation. The results from the four databases were compiled into a new.txt file using R, resulting in a disease-related Venn diagram and a “disease.txt” file. In this process, potential targets were identified aft access er merging the search results and deleting duplicates.

### 2.3. Identification of Intersections between Drug Targets and Disease-Related Genes

To evaluate the intersections between the drug targets and disease-related genes, the Venny 2.1 (http://bioinfogp.cnb.csic.es/tools/venny/index.html, accessed on 1 January 2023) online tool was used for Venn diagram construction to determine the overlap between the active components and disease targets and thus to identify common genes for further analysis.

### 2.4. Construction of the Regulatory Network

For the construction of the Gualou Xiebai Banxia decoction regulatory network, the “allTargets symbol.txt” and “disease.txt” files were placed in one folder. The “net.geneLists.txt”, “net.molLists.txt”, “net.network.txt” and “net.type.txt” files were then obtained using Perl and “net.network.txt” and “net.type.txt” were used for molecular docking. Cytoscape 3.8.0 was then used to integrate the identified active components of the Gualou Xiebai Banxia decoction and its MI targets into a unified network of predicted interactions [24]. In the network, components and genes are linked by lines [25]. Specifically, the “net.network.txt” and “net.type.txt” files were imported into Cytoscape 3.8.0; the gene nodes were selected through “net.genelists.txt” and sorted by “degree”. The layout of the nodes was then transformed to a grid and the size of the nodes was defined. After the selection of the drug component through “net.mollists.txt”, it was defined as a ring. The different ingredients of Gualou Xiebai Banxia decoction were colored differently, with purple representing “gualou”, “xiebai” colored green and “banxia” colored red.

### 2.5. Protein–Protein Interaction (PPI) Network Construction and Core Gene Screening

PPI networks are useful for understanding the functions of specific components and their links to disease and drug effects [26]. Networks were constructed using the Search Tool for the Retrieval of Interacting Genes/Proteins (STRING) database (https://STRING-db.org/, accessed on 1 January 2023) [27]. A confidence level of 0.9 was set to hide individual nodes in the network and the results were saved in TSV format and imported to Cytoscape 3.8.0 for topological analysis and the identification of the hub genes in the network. Specifically, the protein interaction network was first imported into Cytoscape 3.8.0 and the score of each node was calculated by CytoNCA. Nodes were filtered according to the score and nodes with higher scores were selected to rebuild the network and obtain the network core. In the case of multiple network nodes, the network was re-scored to obtain more core hub genes.

### 2.6. Gene Ontology (GO) and Kyoto Encyclopedia of Genes and Genomes (KEGG) Pathway Enrichment Analyses

GO enrichment analysis was performed to examine the functions of the genes in the regulatory network and in which functions they were enriched. To explore the pathways in which the identified genes were involved, KEGG enrichment analysis was performed. The names of the target genes were transformed into Entrez IDs using R [28]. GO and KEGG analyses were conducted in R using cutoff values of False Discovery Rate (FDR) q < 0.05, followed by the selection and graphing of the top 30 items [29]. They used the same packages, which are “colorspace”, “stringi”, “ggplot2”, “BiocManager”, “DOSE’’, “clusterProfiler” and “enrichplot”. KEGG Mapper (https://www.kegg.jp/kegg/mapper/, accessed on 1 January 2023) was used for identifying pathways in which the identified genes were enriched [30].

### 2.7. Molecular Docking

Molecular docking is widely used in structure-based drug discovery [31,32]. The small-molecule ligand and protein receptor files were first prepared. The 2D structures of the TCM components were obtained from PubChem (https://pubchem.ncbi.nlm.nih.gov/, accessed on 1 January 2023) and were converted to 3D structures using ChemOffice. These 3D structures were termed “lig.mol2”. Structures of the hub proteins were downloaded from the Protein Data Bank (http://www.rcsb.org/, accessed on 1 January 2023) and PyMol was used to remove small ligands and water molecules. The structures were then exported and named “rep.pdb”. The “lig.mol2” and “rep.pdb” files were then converted into “.pdbqt” format and the active pockets were identified. Specifically, AutoDockTools was used to hydrogenate receptors and convert ligands to .pbdqt format. The x, y and z dimensions of the active pockets were defined as “40, 40, 40”, respectively, and these parameters were saved. The grid was then exported and saved under the name “grid.gpf”. Finally, docking was performed with AutoDock Vina, using receptor–ligand combinations with the lowest free energy.

## 3. Results

### 3.1. Identification of Active Components of Gualou Xiebai Banxia Decoction

A total of 283 active components were initially identified in the Gualou Xiebai Banxia decoction using the TCMSP database. Of these, 248 components that were unrelated to MI were removed, resulting in 35 components for further analysis; these components are shown in Table 1.

### 3.2. Construction of an Active Component Library and MI Targets

**Figure 2:**
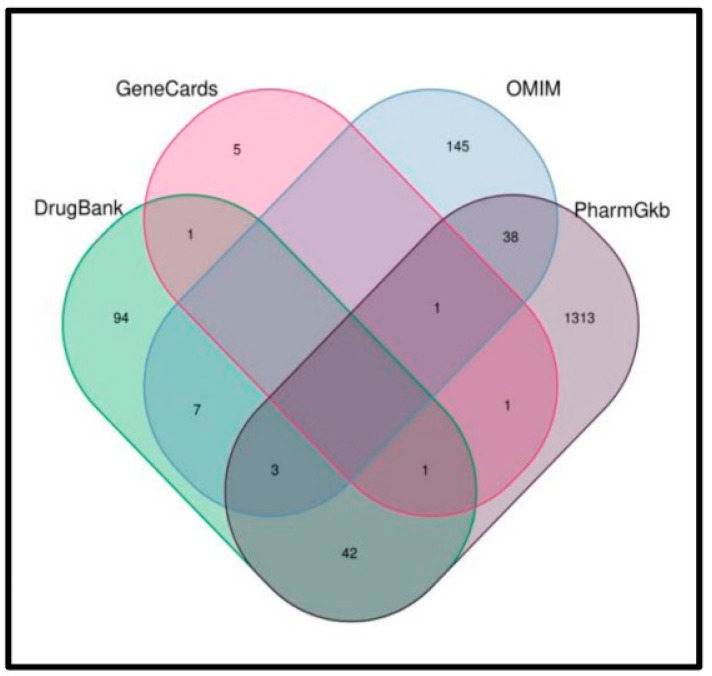
Collection of disease-related genes using GeneCards, OMIM, DrugBank and PharmGkb databases.

**Figure 3:**
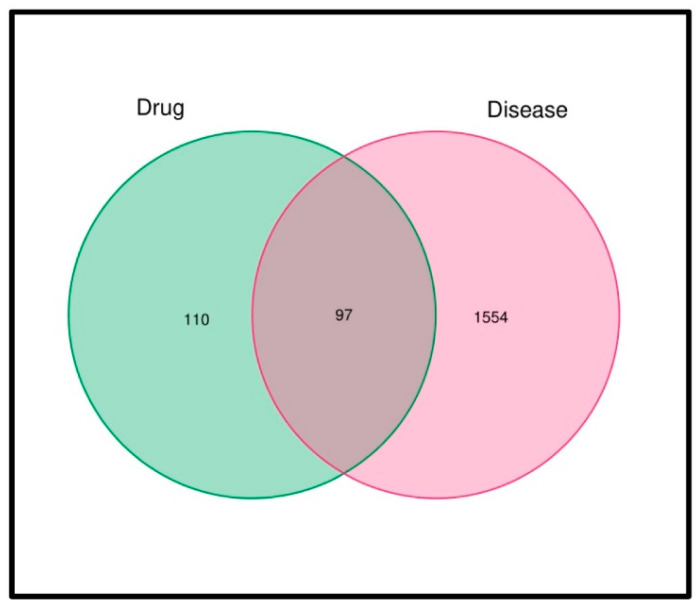
Venn diagram of active ingredients and disease targets. Intersection of drug targets and disease-related genes. The active ingredients refer to “Guolou”, “Xiebai” and “Banxia”.

We used the UniProt database to integrate the information on TCM ingredients and gene symbols in one table and the GeneCards, OMIM, DrugBank and PharmGkb databases to obtain the MI-related targets. This resulted in the identification of 2889 MI-related gene targets. Of these, 1641 were mapped with 207 compound targets, resulting in 97 common target genes (Table 2 and Figure 2 and Figure 3).

### 3.3. Interactive Network of Targets

**Figure 4:**
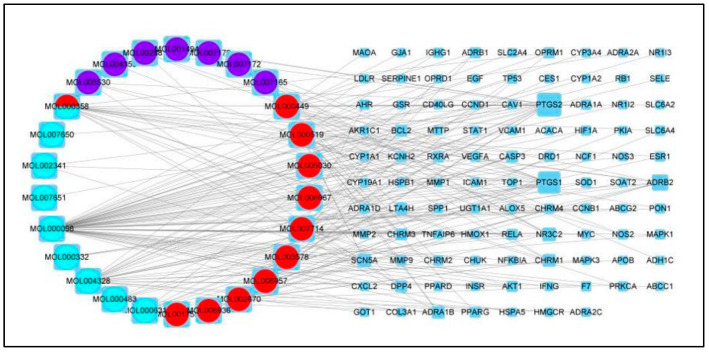
Protein–protein interaction network diagram of Gualou Xiebai Banxia decoction active ingredients and disease targets. The active ingredients refer to “Guolou”, “Xiebai” and “Banxia”. Here, the purple color stands for “Gualou”, the green color for “Xiebai” and the red color for “Banxia”, respectively.

**Figure 5:**
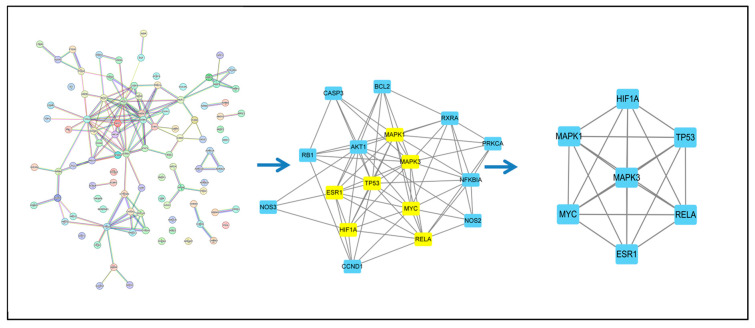
Identification of the PPI network core using Cytoscape 3.8.0. The score of each node is calculated using the cytoscape plug-in (CytoNCA) after selecting a node with a higher score and rebuilding the network to obtain the network core.

PPI networks assist in the exploration of interactions between proteins. In all networks, 97 genes were imported to STRING for PPI network construction and Cytoscape 3.8.0 was used for the identification of the hub target genes and an analysis of the PPI network results (Figure 4). CytoNCA was used to calculate the topology parameters of the nodes using betweenness centrality (BC), closeness centrality (CC), degree centrality (DC), eigenvector centrality (EC), network centrality (NC) and local average connectivity (LAC). The threshold values applied during the first screening were BC > 4.472, CC > 0.072, DC > 3.000, EC > 0.032, NC > 2 and LAC > 1, resulting in 17 nodes and 70 edges. Obviously, this result contained too many nodes and edges and further screening was necessary. The second screening threshold was BC > 6.472, CC > 0.667, DC > 8.000, EC > 0.227, NC > 6.464 and LAC > 4.545, resulting in seven nodes and 21 edges. These seven nodes included HIF1A, MYC, TP53, MAK1, MAK3, ESR1 and RELA (Figure 5). Table 3 shows that these targets have the same “degree”.

### 3.4. Regulatory Network of Active Components

The regulatory network of the TCM compounds was constructed to determine the relationships between the active components and the target genes. The predicted targets of Gualou Xiebai Banxia decoction and the corresponding active components were used to establish a network that was imported into Cytoscape 3.8.0. The network had 123 nodes (26 active components and 97 target genes) (Figure 5). In the figure, the circles on the left represent the active components and the rectangles on the right are the gene targets. Node sizes were determined by the number of associated active components. It can be clearly seen that the Gualou Xiebai Banxia decoction components may target multiple proteins and many TCM ingredients may contain the same monomer compound.

### 3.5. GO and KEGG Pathway Enrichment Analyses

**Figure 6:**
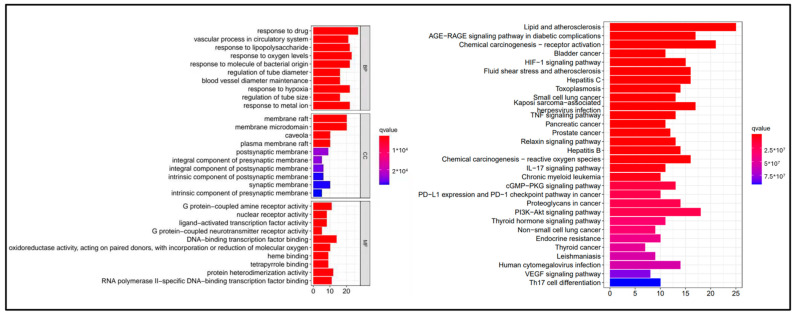
Enriched GO terms and KEGG pathways for 97 target genes.

GO enrichment analysis was conducted to further examine the functions of the intersecting genes. This identified 1767 biological processes (BPs), including “hypoxia reaction”, “drug reaction” and “cell response to lipopolysaccharide”. In the cell components (CC) category, it was found that the proteins were mainly located in the cell membrane. At the same time, the molecular function (MF) category mainly included “binding protein”, “intrinsic protein binding”, “enzyme” and “protein homogenization activity”, amongst others (Figure 6). As shown in Figure 6, gene functions were classified into the GO categories of biological process, cell component and molecular function, which is related to the response to metal ion, response to hypoxia and other biological process functions. This means that Gualou Xiebai Banxia decoction may treat MI by influencing these biological processes, such as response to oxygen levels.

KEGG enrichment analyses of the Gualou Xiebai Banxia decoction target genes were performed using R language. Figure 6 shows the first 30 signal pathways, which involve the PI3K-AKT signaling pathway, IL-17 signaling pathway and TNF signaling pathway and so on. This means that Gualou Xiebai Banxia decoction may treat MI by influencing these pathways, such as the HIF-1 signaling pathway.

It is worth noting that the results of the GO and KEGG analyses were obtained by running the R language and code in conjunction with the Drug_Disease.txt file (Drug_Disease.txt). Interestingly, an analysis of the GO and KEGG results showed that the HIF-1 signaling pathway ranked at the top of the histogram, suggesting that this pathway plays a significant role in MI treatment with the Gualou Xiebai Banxia decoction.

### 3.6. Target Pathway Analysis

**Figure 7:**
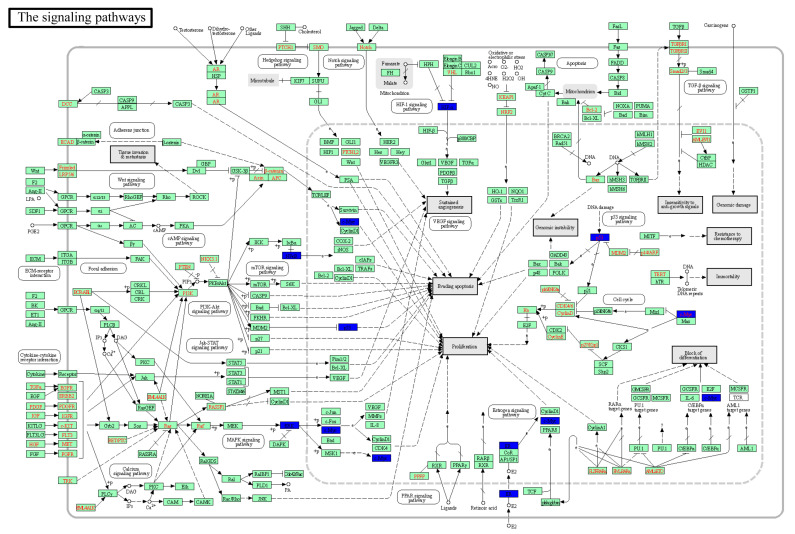
Pathway map of Gualou Xiebai Banxia decoction for MI treatment. Solid arrows indicate activation effects, T arrows indicate inhibition effects and segments indicate activation or inhibition effects. The protein in blue solid box is the pivotal gene of Gualou Xiebai Banxia decoction for MI treatment.

KEGG Mapper was used for the identification of pathway maps, as seen in Figure 7, where the hub targets are colored blue. The identified pathways included the “PI3K signaling pathway”, “Jak signaling pathway”, “HIF-1 signaling pathway” and “mTOR signaling pathway”. As seen in the figure, the hub target genes are mainly involved in cell differentiation and apoptosis, suggesting that the Gualou Xiebai Banxia decoction may regulate several processes and these processes may include specific hub target proteins.

### 3.7. Molecular Docking

**Figure 8:**
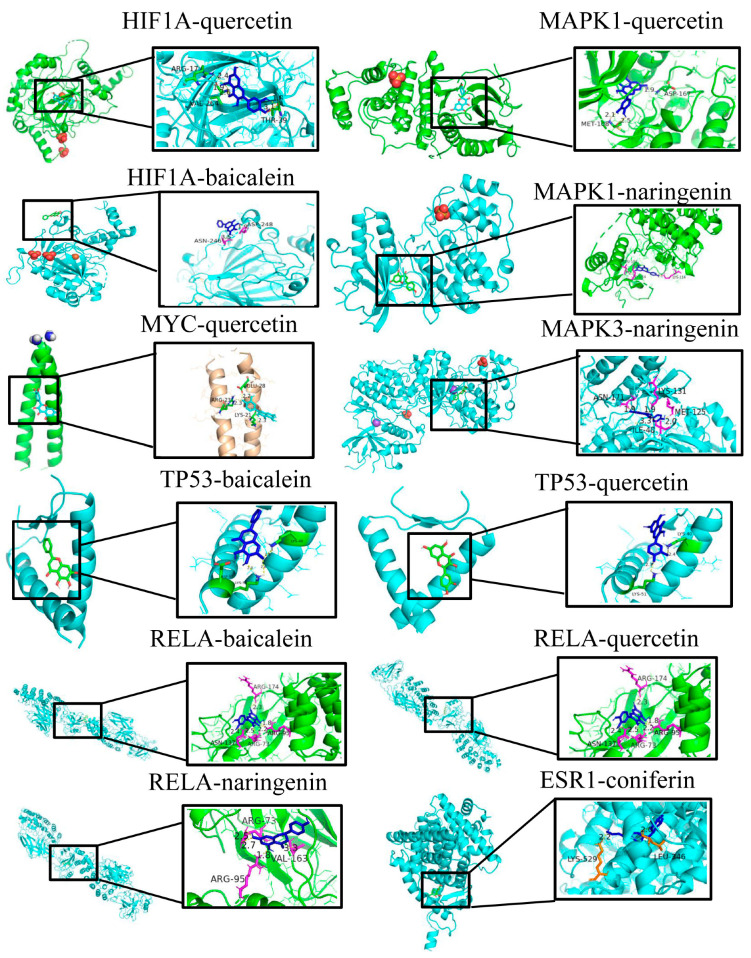
Molecular docking of Gualou Xiebai Banxia decoction active ingredients and hub targets. In this figure, the black box is the active molecule of the drug and the enlarged figure is the combination of the molecule and the protein. These diagrams are predicted based on AutoDock Vina.

Based on the network pharmacology results file (net.network.txt) and the first seven identified protein targets, we identified four compounds, baicalein, quercetin, naringenin and aconitine, which were docked to the first seven identified protein targets. Ligand–receptor docking indicated that the active compounds in the Gualou Xiebai Banxia decoction were likely to interact with the hub proteins and may influence MI treatment through the regulation of HIF1A, MYC, TP53, MAK1, MAPK3, ESR1 and RELA. Details of the four top compounds are shown in Table 4. Table 5 indicates that all the key compounds in the Gualou Xiebai Banxia decoction had a strong affinity for these proteins. The molecular docking results are shown in Figure 8, demonstrating that the active components of Gualou Xiebai Banxia decoction bind to the amino acid residues of hub proteins through hydrogen bonds; for example, quercetin interacts with HIF1A through Arg17, Val264 and Thr39. The lengths of the hydrogen bonds are also shown in Figure 8. Thus, the molecular docking results verified the network pharmacology findings.

## 4. Discussion

Network pharmacology utilizes resemblances between the structures and the documented effects of known drugs and ligands to construct a network of interactions between ligands and targets [33]. This method takes crosstalk between signaling pathways into account, allowing the prediction of multiple interactions that may be used in rational drug design [34,35,36].

Here, we investigated the mechanism of Gualou Xiebai Banxia decoction in treating MI using various techniques, including network pharmacology, pathways analysis and molecular docking between the identified active components and predicted targets. The analysis identified 97 potential target proteins, with PPI network analysis narrowing these down to seven final targets, specifically, HIF1A, MYC, MAPK3, MAPK1, TP53, ESR1 and RELA. Molecular docking (Figure 8) showed that four active components of the decoction, namely, baicalein, quercetin, naringenin and coniferin, can interact directly with these target proteins. Many studies have demonstrated interactions between these active compounds and their targets [37,38,39]. For example, Chao et al. demonstrated that baicalein protects against retinal ischemia through anti-apoptotic effects, as well as through the downregulation of HIF-1α, vascular endothelial growth factor (VEGF) and MMP-9 and upregulation of HO-1 [36]. Our study illustrates that quercetin also interacts with the targets identified here.

GO analysis calculates the hypergeometric distribution relationship to a particular branch of the GO classification, based on the common genes. This analysis is helpful in identifying protein functions and in pharmacological studies [39]. GO annotates genes according to function, while KEGG analysis considers the physiological pathways [40]. The integration of these two procedures allows the identification of specific genes and pathways [41]. KEGG enrichment analysis indicated that the HIF-related pathway, inflammation-related pathways and PI3K/AKT-related pathways may be involved in the treatment of MI by Gualou Xiebai Banxia decoction. Interestingly, RELA, which forms part of the NF-κB pathway, also regulates the inflammatory pathway. In addition, from Figure 8, we can see that the PI3K/AKT pathway indirectly interacts with TP53 and affects the expression of TP53. The MAPK pathway is related to MYC and ESR proteins, while the VEGF signaling identified by KEGG may affect the MAPK pathway [42]. Through the analysis of GO and KEGG, we found that HIF1A, MYC, MAPK, TP53, ESR and RELA were likely targets of the Gualou Xiebai Banxia decoction in treating MI. Importantly, the GO, KEGG and PPI analyses all showed that HIF1A was the most significant of the identified target proteins, suggesting its importance in the action of the Gualou Xiebai Banxia decoction in the prevention or treatment of MI. However, to date, there have been no studies investigating the effects of Gualou Xiebai Banxia decoction on HIF1A, suggesting the usefulness of further investigation of this protein as a novel target of Gualou Xiebai Banxia decoction in cell and animal experiments. In addition, ESR, as one of the main targets, provides a new direction for the study of the mechanism of Gualou Xiebai Banxia decoction. It is worth noting that we have not focused on any sub-datasets for KEGG enrichment analysis, as our previous results have been screened for seven important targets, which we focus on most in our current analysis. However, KEGG results can be used as a reference for further study to demonstrate that these targets or other targets are critical and involved in MI process.

The main purpose of this research was to use network pharmacology prediction and a molecular docking-based strategy to explore the potential mechanism of Gualou Xiebai Banxia decoction in the treatment and prevention of MI. The combination of molecular docking and network pharmacology provides an effective means of studying the effects of Gualou Xiebai Banxia decoction to provide a reference for follow-up research. However, there are some limitations to the present study. It would be worthwhile to conduct additional animal or clinical experiments to verify the results. Issues such as the TCM dosage, safety and its effects after administration also require further investigation.

In this paper, we used AutoDock Vina for molecular docking, which has a relatively high average accuracy of binding mode prediction and easy operation. It successfully simulates the binding of large-molecule proteins and small-molecule ligands. We focused on network pharmacology combined with molecular docking. Network pharmacology emphasizes holistic systems and biological networks when analyzing the laws of molecular association between drugs and therapeutic targets. By focusing on drug research, it allows for an analysis of the complexity of TCM formulations, together with an explanation of the overall mechanisms of action and the provision of new scientific and technological support for drug development. With the increasing influence and applications of the big data era, network pharmacology faces both significant development opportunities and challenges [43]. There are still many problems in current network pharmacology research, such as uneven study quality, a lack of standardized data and insufficient scientific validation. Moreover, some of the predicted targets and pathways may have undesirable effects on the drug safety, such as dizziness, headache and nitrate-related tolerance [44]. Therefore, the optimal dose and duration of the compound require further investigation before clinical application. Thus, the establishment of rigorous, scientific and uniform criteria for the evaluation of network pharmacology is urgently needed for the further development of network pharmacology research [43].

To summarize, Gualou Xiebai Banxia decoction contains many active components that interact with specific targets to influence downstream pathways, leading to the treatment of MI. The results showed that baicalein, quercetin, naringenin and coniferin were the key active compounds in Gualou Xiebai Banxia decoction and suggested that they may play significant roles in MI treatment. In addition, HIF1A, MYC, MAPK3, MAPK1, TP53, ESR1 and RELA were identified as key target proteins and, of these, HIF1A was the most significant. This article is the first to systematically explore the possible targets and mechanisms of Gualou Xiebai Banxia decoction in the treatment of MI. It provides a new theoretical basis for exploring therapeutic drugs and their mechanisms for the treatment of MI. However, the precise roles and mechanisms of action of the active components of Gualou Xiebai Banxia decoction in the treatment of MI have not been fully explained and verified, suggesting directions for future research.

## Figures and Tables

**Table 1:** The effective ingredients of Gualou Xiebai Banxia decoction.

Drug Name	Mol ID	Molecule Name	OB (%)	DL
Gualou	MoL001494	Mandenol	42	0.19
Gualou	MoL002881	Diosmetin	31.14	0.27
Gualou	MoL004355	Spinasterol	42.98	0.76
Gualou	MoL005530	Hydroxygenkwanin	36.47	0.27
Gualou	MoL 006756	Schottenol	37.42	0.75
Gualou	MoL007156	10α-cucurbita-5,24-diene-3β-ol	44.02	0.74
Gualou	MoL007171	5-dehydrokarounidiol	30.23	0.77
Gualou	MoL007172	7-oxo-dihydrokaro-unidiol	36.85	0.75
Gualou	MoL007175	karounidiol 3-o-benzoate	43.99	0.5
Gualou	MoL007179	Linolenic acid ethyl ester	46.1	0.2
Gualou	MoL007180	vitamin-e	32.29	0.7
Xiebai	MoL001973	Sitosteryl acetate	40.39	0.85
Xiebai	MoL002341	Hesperetin	70.31	0.27
Xiebai	MoL000332	n-coumaroyltyramine	85.63	0.2
Xiebai	MoL000358	β-sitosterol	36.91	0.75
Xiebai	MoL004328	naringenin	59.29	0.21
Xiebai	MoL000483	(Z)-3-(4-hydroxy-3-methoxy-phenyl)-N-[2-(4-hydroxyphenyl)ethyl]acrylamide	118.35	0.26
Xiebai	MoL000631	coumaroyltyramine	112.9	0.2
Xiebai	MoL007640	macrostemonoside e_qt	35.26	0.87
Xiebai	MoL007650	PGA(sup 1)	43.98	0.25
Xiebai	MoL007651	Prostaglandin B1	40.21	0.25
Xiebai	MoL000098	quercetin	46.43	0.28
Banxia	MoL001755	24-Ethylcholest-4-en-3-one	36.08	0.76
Banxia	MoL002670	Cavidine	35.64	0.81
Banxia	MoL002714	baicalein	33.52	0.21
Banxia	MoL002776	Baicalin	40.12	0.75
Banxia	MoL000358	β-sitosterol	36.91	0.75
Banxia	MoL000449	Stigmasterol	43.83	0.76
Banxia	MoL005030	gondoic acid	30.7	0.2
Banxia	MoL000519	coniferin	31.11	0.32
Banxia	MoL006936	10,13-eicosadienoic	39.99	0.2
Banxia	MoL006937	12,13-epoxy-9-hydroxynonadeca-7,10-dienoic acid	42.15	0.24
Banxia	MoL006957	(3S,6S)-3-(benzyl)-6-(4-hydroxybenzyl)piperazine-2,5-quinone	46.89	0.27
Banxia	MoL003678	Cycloartenol	38.69	0.78
Banxia	MoL006967	β-D-Ribofuranoside, xanthine-9	44.72	0.21

Abbreviations: DL, drug similarity; OB, oral bioavailability.

**Table 2:** The common targets of Gualou Xiebai Banxia decoction and MI.

Targets	Full Name
NR3C2	nuclear receptor subfamily 3 group C member 2
PTGS1	prostaglandin-endoperoxide synthase 1
CHRM3	M3 muscarinic acetylcholine receptor
KCNH2	K(+) voltage-gated channel subfamily H member 2
CHRM1	Epigenetic regulation of cholinergic receptor M1
ADRB1	adrenergic receptor β 1
SCN5A	cardiac sodium channel
PTGS2	Prostaglandin-Endoperoxide Synthase 2
ADRA2C	α-2C-adrenergic receptor gene
CHRM4	cholinergic receptor, muscarinic 4
RXRA	retinoid X receptor protein
OPRD1	opioid receptor delta 1
ADRA1B	α-adrenergic receptor-1b
ADRB2	beta2-adrenergic receptor
ADRA1D	α(1) adrenoreceptor subtype D
OPRM1	opioid Receptor Mu 1
DRD1	dopamine receptors 1
SLC6A4	solute carrier family 6 member 4
F7	genes of factor VII
DPP4	dipeptidyl peptidase-4
RELA	NFkappaB-p65
AKT1	protein kinase B
VEGFA	vascular endothelial growth factor A
BCL2	B-cell lymphoma 2
MMP9	gelatinase matrix metalloproteinase 9
CASP3	Caspase-3
TP53	tumor protein p53
HIF1A	hypoxia-inducible factor 1alpha
CCNB1	cyclin B1
AHR	aryl hydrocarbon receptor
ADRA1A	α-1A-adrenoreceptor
CHRM2	cholinergic muscarinic 2 receptor
PRKCA	protein kinase C α
PON1	Paraoxonase-1
ADH1C	Alcohol dehydrogenase 1C
IGHG1	immunoglobulin heavy constant γ 1
ADRA2A	adrenergic receptor α-2A
SLC6A2	solute carrier family 6 member 2
LTA4H	leukotriene A4 hydrolase
MAOA	Monoamine oxidase A
ESR1	estrogen receptor 1
PPARG	peroxisome proliferator-activated receptor γ
NOS2	nitric oxide synthase
MAPK3	mitogen-activated protein kinase 3
MAPK1	mitogen-activated protein kinase 1
LDLR	Low-density lipoprotein receptor
SOD1	superoxide dismutase 1
MTTP	microsomal triglyceride transfer protein
APOB	apolipoprotein B
HMGCR	3-Hydroxy-3-methylglutaryl coenzyme A reductase
CYP19A1	cytochrome P4501A1
UGT1A1	uridine diphosphate glucuronosyltransferase 1A1
GSR	galvanic skin response
ABCC1	multidrug resistance protein 1
SOAT2	sterol O-acyltransferase 2
AKR1C1	Aldo-keto-reductases 1C1
GOT1	glutamate oxaloacetate transaminase 1
CES1	Carboxylesterase 1
PKIA	Protein Kinase Inhibitor α
CCND1	Cyclin D1
MMP2	matrix metalloproteinase 2
EGF	epidermal growth factor
RB1	retinoblastoma susceptibility gene
TNFAIP6	TNFalpha-stimulated gene-6
NFKBIA	nuclear factor-kappa-B inhibitor-α
TOP1	topoisomerase I
MMP1	matrix metallopeptidase 1
STAT1	signal transducer and activator of transcription 1
HSPA5	Heat shock 70 kDa protein 5
ACACA	acetyl-CoA carboxylase α
HMOX1	heme oxygenase 1
CYP3A4	cytochrome P450 3A4
CYP1A2	cytochrome P450 1A2
CAV1	Caveolin-1
MYC	c-Myc
GJA1	Gap Junction Protein α 1
CYP1A1	cytochrome P450 1A1
ICAM1	intercellular adhesion molecule 1
SELE	E-selectin
VCAM1	vascular cell adhesion molecule 1
NOS3	nitric oxide synthase 3
HSPB1	HSP β-1
NR1I2	nuclear receptor subfamily 1 group I member 2
SERPINE1	serine protease inhibitor clade E member 1
IFNG	interferon γ
ALOX5	arachidonate 5-lipoxygenase
NCF1	neutrophil cytosolic factor 1
ABCG2	ATP-binding cassette (ABC) superfamily G member 2
SLC2A4	solute carrier family 2 member 4
COL3A1	Collagen type III α 1
CXCL2	CXC motif chemokine ligand 2
NR1I3	constitutive androstane receptor
INSR	insulin receptors
PPARD	peroxisome proliferator-activated receptor β/delta
CHUK	conserved helix-loop-helix ubiquitous kinase
SPP1	secreted phosphoprotein 1

**Table 3:** The centrality measures core targets of Gualou Xiebai Banxia decoction and MI.

Target Gene Symbol	BC	CC	DC	EC	LAC	NC
ESR1	0	1	6	0.377964467	5	6
TP53	0	1	6	0.377964616	5	6
MYC	0	1	6	0.377964497	5	6
MAPK1	0	1	6	0.377964497	5	6
RELA	0	1	6	0.377964497	5	6
HIF1A	0	1	6	0.377964497	5	6
MAPK3	0	1	6	0.377964497	5	6

Abbreviations: BC, betweenness centrality; CC, closeness centrality; DC, degree centrality; EC, eigenvector centrality; NC, network centrality; LAC, local average connectivity.

**Table 4:** The basic information of the top four compounds.

Molecule Name	Molecular Formula	Structure
Baicalein	C_15_H_10_O_5_	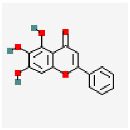
Quercetin	C_15_H_10_O_7_	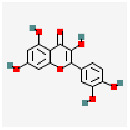
Naringenin	C_15_H_12_O_5_	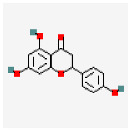
Coniferin	C_16_H_22_O_8_	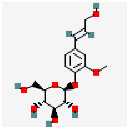

**Table 5:** The binding energy of top four compounds.

	Baicalein	Quercetin	Naringenin	Coniferin
HIF1A docking score (kcal/mol)	−4.7	−7.4	\	\
MYC docking score (kcal/mol)	\	−5.6	\	\
TP53 docking score (kcal/mol)	−6.3	−6.3	\	\
MAK1 docking score (kcal/mol)	\	−8.5	−8.4	\
MAK3 docking score (kcal/mol)	\	\	−8.7	\
RELA docking score (kcal/mol)	−6.9	−7.7	−7.4	\
ESR1 docking score (kcal/mol)	\	\	\	−7.3

## Data Availability

We have presented all our main data in the form of figures and tables. The datasets supporting the conclusions of this article are included within the article.

## Abbreviations

MI	Myocardial infarction
TCM	Traditional Chinese medicine
CVDs	Cardiovascular diseases
AMI	Acute myocardial ischemia
T2DM-AMI	Type II diabetes with acute myocardial ischemia
OS	Oxidative stress
PI3K	Phosphatidyl inositol 3-kinase
AKT	Threonine protein kinase B
TCMSP	Traditional Chinese Medicine Systems Pharmacology Database and Analysis Platform
OB	Oral bioavailability
DL	Drug-likeness
PPI	Protein–protein interaction
BC	Betweenness centrality
CC	Closeness centrality
DC	Degree centrality
EC	Eigenvector centrality
LAC	Local average connectivity
NC	Network centrality
BP	Biological processes
CC	Cell components
MF	Molecular function
AGE-RAGE	Advanced glycation end-RAGE
IL-17	Interleukin (IL)-17
TNF	Tumor necrosis factor
HIF-1	Hypoxia-inducible factor 1
Jak	Janus kinases
mTOR	Mammalian/mechanistic target of rapamycin
MYC	c-Myc
TP53	Wild-type (WT) human p53
MAK1	Mitogen-activated protein kinase 1
MAK3	Mitogen-activated protein kinase 3
ESR1	Estrogen receptor 1
RELA	NFkappaB-p65
NF-κB	NF-kappaB
VEGF	Vascular endothelial-derived growth factor
